# Reallocation of water resources according to social, economic, and environmental parameters

**DOI:** 10.1038/s41598-021-96680-2

**Published:** 2021-09-01

**Authors:** Alireza Rezaee, Omid Bozorg-Haddad, Xuefeng Chu

**Affiliations:** 1 Department of Irrigation and Reclamation Engineering, Faculty of Agriculture Engineering and Technology, College of Agriculture and Natural Resources, University of Tehran, Karaj, Alborz Iran; 2 Department of Civil and Environmental Engineering, North Dakota State University, Dept. 2470, Fargo, ND 58108-6050 USA

**Keywords:** Climate sciences, Ecology, Environmental sciences, Environmental social sciences, Hydrology, Natural hazards, Energy science and technology, Engineering, Materials science, Mathematics and computing

## Abstract

Population growth, urbanization, and industrial development have significantly increased water demands in many countries, raising the concerns about water resources sustainability to meet the needs of humans and the environment. Furthermore, the economy-oriented allocation of water resources has caused many socio-environmental problems. The main goal of this study is to develop a system dynamics modeling framework that integrates economic, social, and environmental dimensions for the decision of water resources allocation. The Technique for Order of Preference by Similarity to Ideal Solution (TOPSIS) is used to rank modeling scenarios and identify the best strategy for water allocation. In the application to East Azerbaijan province of Iran, six industry groups (including chemical, food and beverage, non-metal, machinery and equipment, metal, and textile), thirteen water allocation scenarios, and five criteria (including profit index, employment index, return of surface water, groundwater sustainability index, and total allocated water) were considered. The TOPSIS results showed that in the best scenario most water was allocated to the non-metal industry with a relative distance of 0.63 to the ideal solution. On the other hand, the current water allocation scenario ranked seventh, indicating that significant improvements are required to take into account the social, economic, and environmental factors for optimal reallocation of water resources among different industry users.

## Introduction

The current water crisis has increased attention to the effective management of water resources. Water is crucial to the sustainable development of societies, which involves the economic, social, and environmental sectors^1^. On the other hand, the expansion of economic and agricultural activities in recent decades has exacerbated the problem of water scarcity and increased conflicts and political tensions, which highlight the need for sustainable development and optimization of resource allocations. Iran is one of the arid and semi-arid countries. It is of particular importance to pay attention to the social and environmental aspects of water resources management and analyze their relationships.

Allocation of water resources among different sectors has been an important management issue. Many studies have been conducted to maximize the profit of water allocations. Babel et al.^2^ developed an integrated model for the optimal allocation of reservoir water. Their model included three sub-models for evaluation of reservoir performance, economic analysis, and water allocation with linear objective functions of maximizing consumer satisfaction and net profit. Although the environment water need was considered, it was not considered in the objective function of their optimization model. Banihabib et al.^3^ developed a nonlinear model for managing water allocation to increase the net profit from industry and services and their results showed that the optimization model increased water productivity, reduced water demand dissatisfaction, and preserved agricultural products. Similar studies have also been conducted to optimize water management with an objective function of maximizing economic benefits (e.g.,^4–9^).

Water resources and social parameters have a two-way relationship, as they influence each other^1^. Keshavarz et al.^10^ studied the effects of water scarcity and drought on the households of two villages in Fars province of Iran, including the reduction of their income, food insecurity, reduction of their health index, reduction of access to educational facilities, stress, migration, and despair. Dean et al.^11^ performed a statistical analysis of using alternative water sources that focused on rainwater extraction, desalination, and recycled water in Australia, and showed indirect effects on employment status, life satisfaction, and discussion within families. Scanlon et al.^12^ examined the effects of water availability on the life of the residents (e.g., livelihood and employment) in Africa. In addition, Popovic et al.^13^, El-Gafy^14^, Bui et al.^15^, and Li et al.^16^ also examined different social-economic and water parameters and their relationships.

In water resources allocation, most decision-makers seek to maximize the economic benefits of water resources, and less attention has been paid to sustainable development. The direct and indirect influences of water allocation on sustainability have emphasized the need to pay more attention to the social and environmental aspects of water resources allocation. Ahmadi et al.^17^ developed a multi-objective water resources management model with economic, social, and environmental objectives related to water quantity and quality, downstream water demands, agricultural production, and employment to determine land use and water allocation. Tu et al.^18^ presented an optimization model for solving agricultural water allocation problems in China to maximize economic benefits and minimize environmental pollution. Their results showed that increasing economic profits did improve the general well-being of the society, but increased the potential of pollution, which led to a decrease in social satisfaction.

Some studies have been conducted to account for the economic, social, and environmental factors in water resources allocation. Kelly et al.^19^ reviewed five common approaches that had the capacity to integrate knowledge by accommodating multiple issues in models. In the related studies, the impacts of agricultural, industrial, urban, and environmental water consumptions on social, economic, and environmental factors were considered, but the relations within the industry sector have not been carefully considered. The difference of the influences of each group of industries on socio-economic and environmental evaluation indicators for water allocation is still unclear^20,21^. This study aims to fill this gap.

The objective of this study is to develop a system dynamics modeling framework to integrate economic, social, and environmental dimensions for the allocation of water resources to industries. The Technique for Order of Preference by Similarity to Ideal Solution (TOPSIS) is used to rank modeling scenarios and identify the best strategy for water allocation. The application in East Azerbaijan province of Iran involves six industry groups (including chemical, food and beverage, non-metal, machinery and equipment, metal, and textile), thirteen water allocation scenarios, and five evaluation criteria^22^, including profit index (the total amount of industry profits from the sale of products, unit: Billion Rials), employment index (the number of people employed in the industrial sector, unit: Person), return of surface water (the amount of water returned from industries to surface water sources, unit: mcm), groundwater sustainability index (the difference between the amounts of water consumed and returned to groundwater resources, unit: mcm), and total allocated water (the total amount of water allocated to industries from surface and groundwater resources, unit: mcm).

## Study region

**Figure 1:**
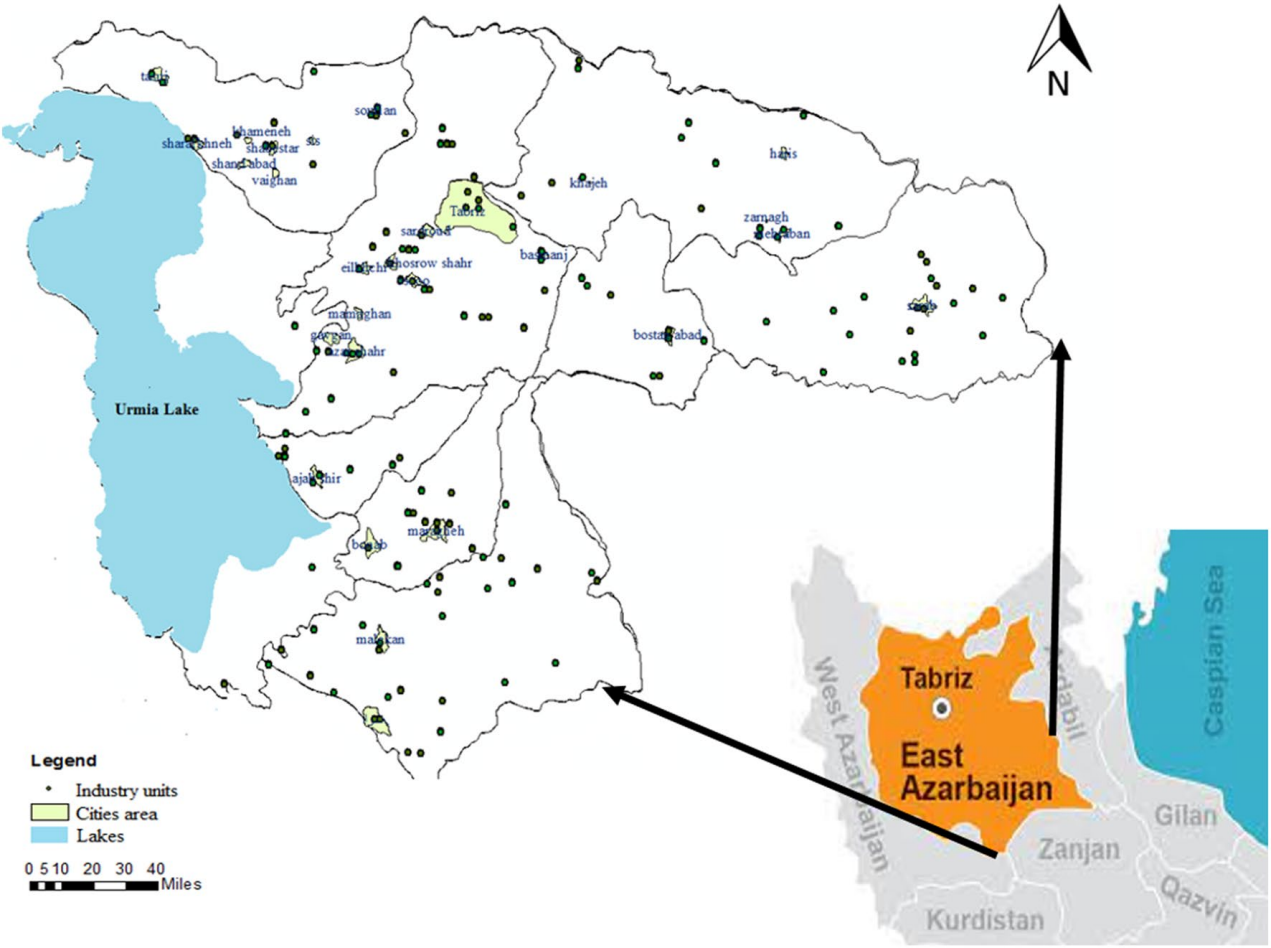
Location of the study area and the distribution of industry units (ArcGIS 10.7.1).

Tabriz is the fourth largest city in Iran and the most important city in the Urmia Lake basin, which is located in Northwestern Iran (Fig. 1) and is one of the six main basins in Iran with an area of 51,876 km^2^. It includes about half of West Azerbaijan province, a large part of East Azerbaijan province, and a portion of Kurdistan province.

**Figure 2:**
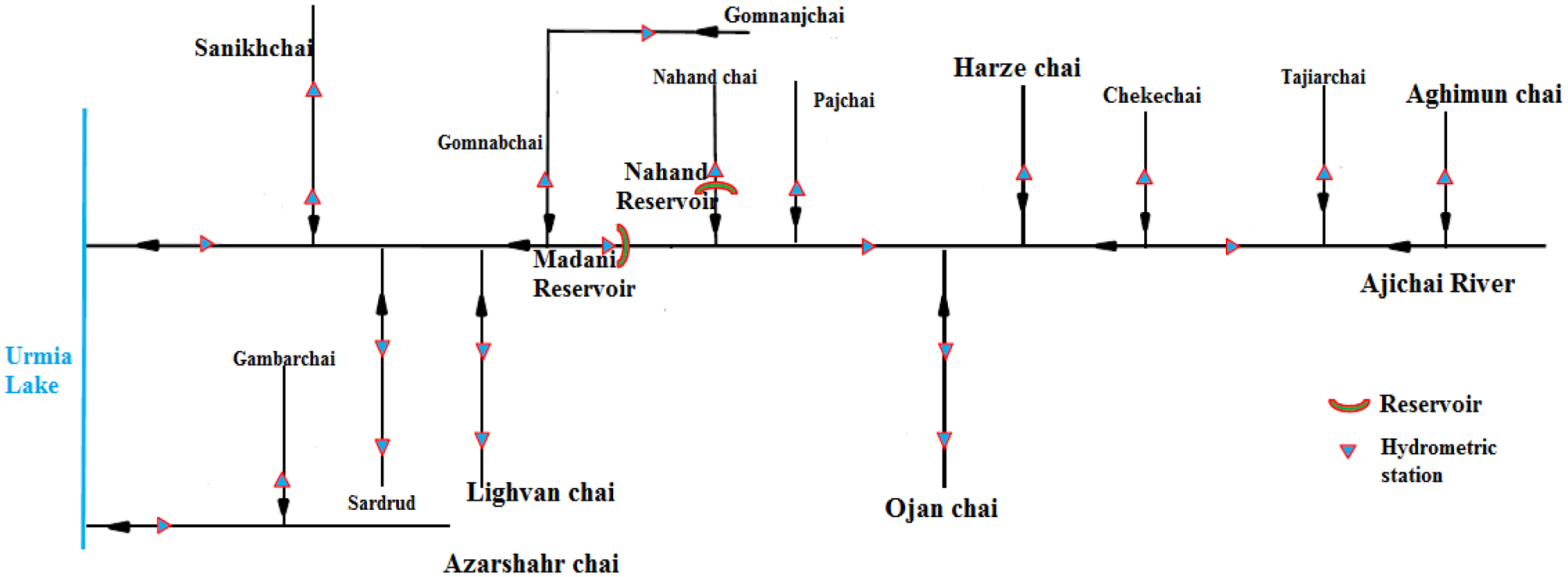
Rivers and their connections to Urmia Lake and the hydrometric stations.

Aji Chai is the major river in the Urmia Lake basin. The length of this river is 265 km, and its catchment area is about 9200 km^2^. The cities of Tabriz, Azarshahr, Sarab, Bostan Abad, Harris, and Osko are the main urban areas of this basin. The drainage network of the Urmia Lake basin and the hydrometric stations are shown in Fig. 2.

This study focuses on East Azarbaijan province of Iran, which includes about 4500 active scattered industrial units in the Urmia Lake basin. These industries across a 64-ha area employ a total of 19,000 people. According to their water consumption, the industries of this province are classified into six categories: food and beverage, non-metal, textile, chemical, machinery and equipment, and metal. The data related to the amount of water consumed by these industries, the number of employees, the amount of produced effluent, and their profits were acquired from the National Statistics Center of Iran^23^.

## System dynamics

System dynamics introduced by Forrster (1974) is a powerful simulation approach for solving, supporting, and decision-making complex problems such as basin management^24^, reservoir operation and flood management^25–27^, and management of a dynamic water resources-socioeconomic-environmental system^28^. This approach involves computer simulations for a complex dynamic system with feedback process inclusion and provides a way to model the actual behavior of the system.

The increasing use of surface water and groundwater resources due to the increased water demands has led to a decreased trend in the available water resources in many regions. To integrate water resources systems and adopt the sustainable development policies to mitigate the related water crisis, a support tool is needed to make the best decision for water allocation. Considering the variability of the relevant parameters over time and the impacts on other non-aqueous parameters in an area, the system dynamics can be a good option if sufficient knowledge of causal and systemic loops of the system can be used to simulate different policies for decision-makers before implementation.

**Figure 3:**
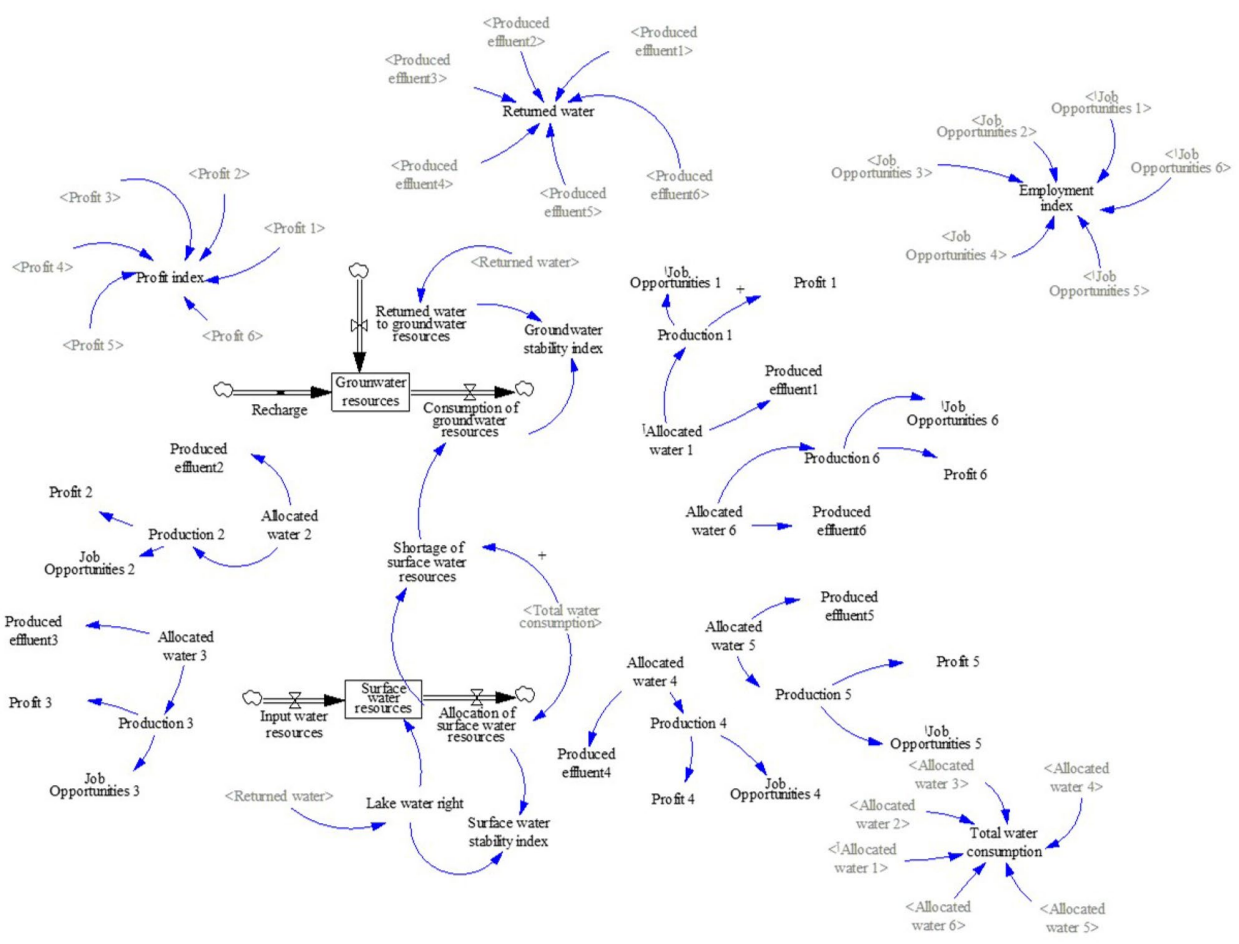
Stock and flow diagram of industrial water allocations of this study.

In this study, by using the data of 2016, the relationships of the industrial consumption of surface water and groundwater resources, industrial production rate, industrial profit rate, the number of jobs created, as well as production effluent and return water to surface and subsurface systems were analyzed. Using the VENSIM software, a series of simulations were performed for different industrial water allocation scenarios. Based on the identified relationships, the general stock-flow structure was developed (Fig. 3). As shown in Fig. 3, surface water and groundwater resources were considered as two stock and flow diagrams and two main loops were created. Some of the causal relationships extracted are described in the following sections.

### Water consumption and industrial production

**Figure 4:**
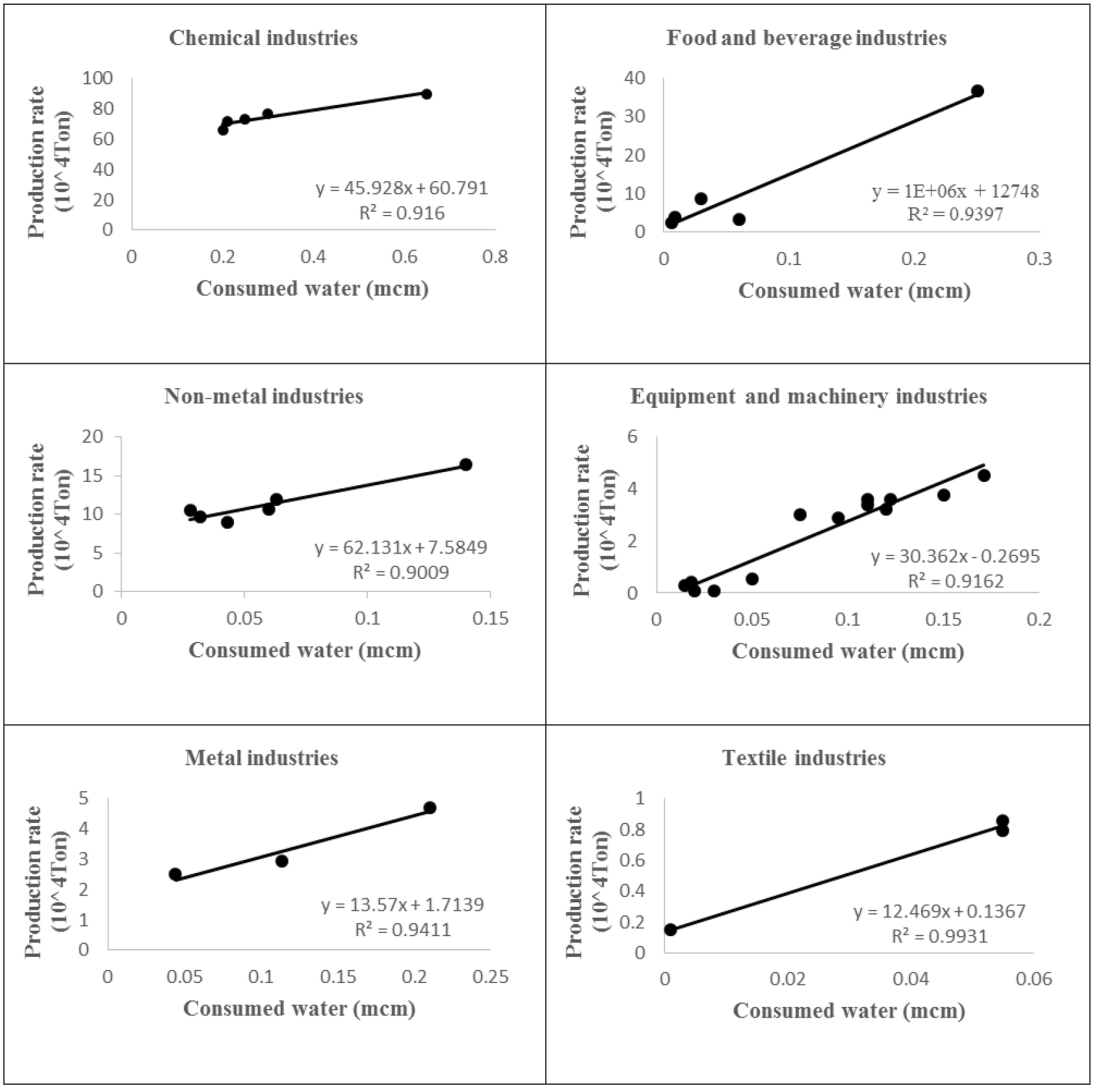
Correlation between consumed water and production of the industries.

Water is one of the main factors in the production of agricultural and industrial products. Thus, the production is directly and indirectly dependent on water resources. In this study, six groups of industries were correlated with their water consumptions. The correlation relationships were used to calculate the related parameters for a set of modeling scenarios generated by different percentages of water consumption. The correlation relationships between the consumed water and the production of the industries are shown in Fig. 4. All the original data were obtained from the National Statistics Center of Iran^29^.

### Production and employment

**Figure 5:**
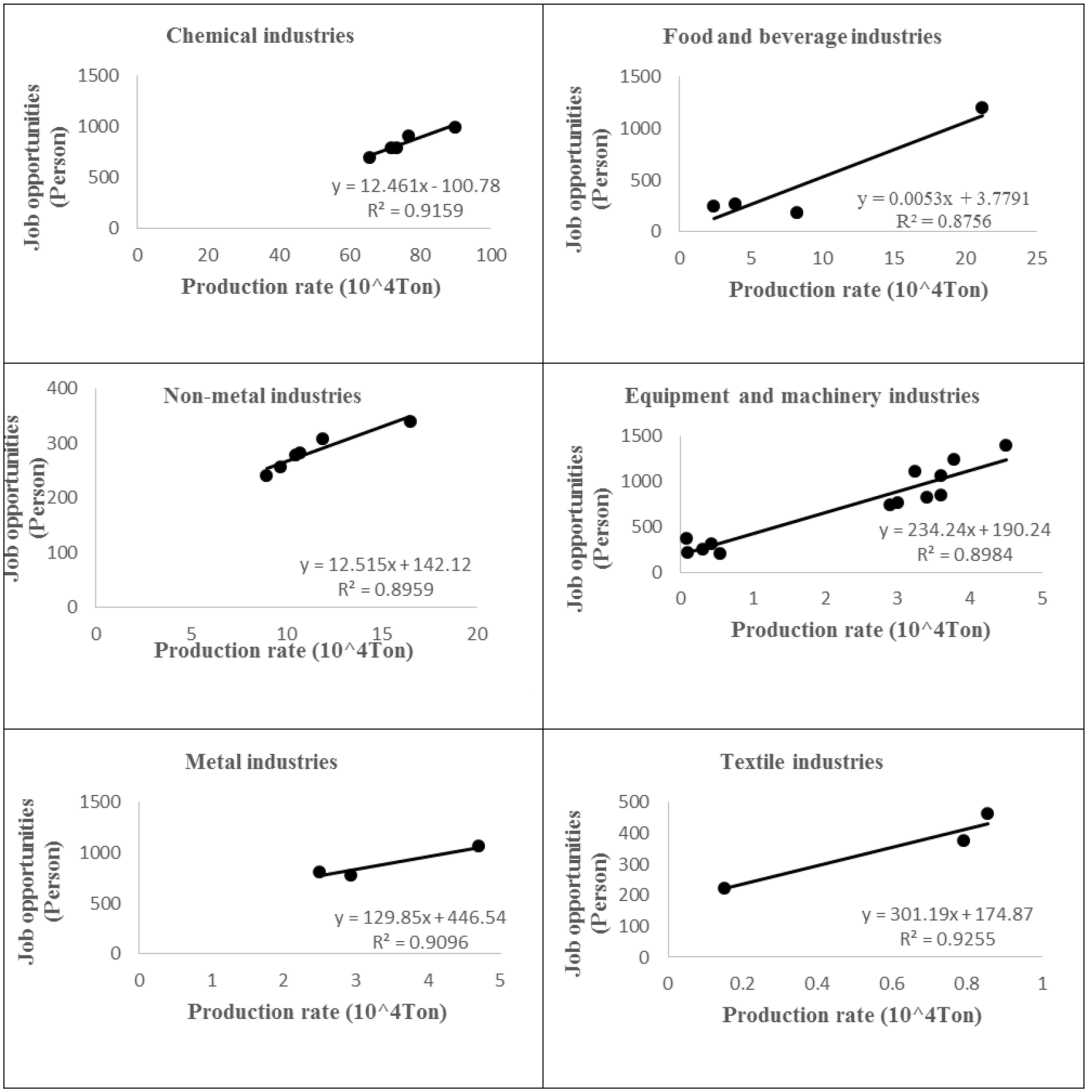
Correlation between production and job opportunities (employment) of the industries.

The employment rate in the industrial sector is higher than that in the agricultural sector, and according to Rezaee et al.^30^, the production and the job opportunities created had a good correlation. Based on the amount of production of each industry group and the amount of its employment created, the relationship between production and employment was determined. The production-employment correlation relationships for all industries are shown in Fig. 5. These original data were also obtained from the National Statistics Center of Iran^29^.

### Production effluent

During the production using the allocated water, the industries also produce wastewater or effluent. The amount of the effluent from a specific industry varies. Using the data published by the Iran Environment Organization^23^, the percentages of effluent produced by the six industries were calculated (Table 1). As shown in Table 1, the chemical industry had the highest effluent than the others.

**Table 1:** Percentages of industrial effluent.

Industry group	Percentage of produced wastewater out of the consumed water (%)
Chemical	23.98
Food and beverage	6.57
Non-metal	8.70
Equipment and machinery	10.34
Metal	7.14
Textile	8.33

### Industrial water allocation scenarios

In this study, the percentages of the current water allocations for the six industries in the province (Ministry of Energy,^31^) were considered as the basic scenario. According to the current water allocations in Table 2, the highest and lowest water allocation percentages are 28.09% and 1.93% for the chemical and textile industries, respectively. To create different water allocation scenarios that cover possible modification modes to analyze the sensitivity of different allocation options, percentage changes in water allocations were made for the industries compared to the current water allocations (i.e., basic scenario). The amount of production of the selected industries depends on the amount of allocated water, and therefore the change in the percentage of allocated water is limited. In this study, to analyze the sensitivity of the industries and to obtain reasonable changes in the values of the five selected evaluation criteria (i.e., profit index, employment index, return of surface water, groundwater sustainability index, and total allocated water). Although 2%, 5%, and 10% changes in the allocated water were applied, there were no significant changes in the evaluation indicators compared to the basic scenario. So, a percentage change of 20% in the allocated water was selected to create different modeling scenarios. It should be noted that further changes were subject to the limitations of available water resources and industrial equipment capacities for more production. Thus, in one scenario the amount of water allocated to the first industry was increased by 20%, while the amount of water allocated to the other five industries remained unchanged. This allocation fashion continued for all six industries. The generated scenarios are shown in Table 3.

**Table 2:** Water allocations for the basic scenario.

Industry group	Allocated water (%)
Chemical	28.09
Food and beverage	10.47
Non-metal	16.28
Equipment and machinery	18.95
Metal	24.25
Textile	1.93

**Table 3:** Thirteen modeling scenarios and their water allocations to six industries.

Scenario number	Scenario description	Industry 1 (mcm)	Industry 2 (mcm)	Industry 3 (mcm)	Industry 4 (mcm)	Industry 5 (mcm)	Industry 6 (mcm)
1	Basic scenario	1.61	0.6	0.933	1.086	1.39	0.111
2	20% increase in industry 1	1.932	0.6	0.933	1.086	1.39	0.111
3	20% reduction in industry 1	1.288	0.6	0.933	1.086	1.39	0.111
4	20% increase in industry 2	1.61	0.72	0.933	1.086	1.39	0.111
5	20% reduction in industry 2	1.61	0.48	0.933	1.086	1.39	0.111
6	20% increase in industry 3	1.61	0.6	1.1196	1.086	1.39	0.111
7	20% reduction in industry 3	1.61	0.6	0.7464	1.086	1.39	0.111
8	20% increase in industry 4	1.61	0.6	0.933	1.3032	1.39	0.111
9	20% reduction in industry 4	1.61	0.6	0.933	0.8688	1.39	0.111
10	20% increase in industry 5	1.61	0.6	0.933	1.086	1.668	0.111
11	20% reduction in industry 5	1.61	0.6	0.933	1.086	1.112	0.111
12	20% increase in industry 6	1.61	0.6	0.933	1.086	1.39	0.1332
13	20% reduction in industry 6	1.61	0.6	0.933	1.086	1.39	0.0888

Industries 1–6 denote chemical, food and beverage, non-metal, equipment and machinery, metal, and textile, respectively.

## TOPSIS

TOPSIS^32^, a multi-criteria decision-making method has been successfully implemented in various studies, and the results have revealed its great potential as an MADM method (e.g.,^33,34^). After simulating the generated scenarios and calculating the values of the five evaluation indicators, TOPSIS was used to rank the scenarios and select the best one. Note that by using the multi-criteria decision-making method, it is no longer necessary to convert the social and environmental criteria to the corresponding economic equivalents. Instead, different quantitative and qualitative criteria can be directly used to prioritize and select options. In the TOPSIS, the best option is selected based on the shortest distance from the ideal option and the longest distance from the worst option^35^. The ideal positive option is the one that has the highest possible utility and may not necessarily exist externally. The modeling procedures and steps are briefly explained as follows.

### Weighting of indicators

In the TOPSIS, the decision matrix must be weighed. In this study, Shannon's entropy method was used. As an auxiliary method, by forming a matrix with rows of research options and columns of research criteria, the weights of the criteria were calculated. Note that in the entropy weighting method, more weights were given to the indicators with higher variability.

*Step 1* Determine the entropy value of each indicator by^36^:

1 $$ E_{j} = - \frac{1}{Ln(m)}\left( {\sum\limits_{i = 1}^{m} {r_{i,j} Ln(r_{ij} )} } \right),\quad j = 1, \ldots ,n $$

where *r*_*i,j*_ is the value of the *i*th alternative with regard to the *j*th criterion in the decision matrix; *m* is the total number of feasible alternatives; and *n* is the total number of criteria.

*Step 2* Determine the uncertainty value of each indicator by:

2 $$ d_{j} = 1 - E_{j} $$

where *d*_*j*_ is the uncertainty of indicator *j*.

*Step 3* Calculate the weight of each decision variable by:

3 $$ W_{j} = \frac{{d_{j} }}{{\sum\nolimits_{j = 1}^{n} {d_{j} } }} $$

where *W*_*j*_ denotes the importance value (or weight) of the *j*th criterion.

### Computing the relative closeness to the ideal solution

The following steps were implemented to determine the relative closeness of each alternative to the ideal solution.

*Step 1* Calculate the normalized decision-making matrix by multiplying the normalized decision matrix by the vector of the specified coefficients:

4 $$ V = \left( {\begin{array}{*{20}c} {w_{1} \times r_{11} } & \ldots & {w_{n} \times r_{1n} } \\ \vdots & \ddots & \vdots \\ {w_{1} \times r_{m1} } & \ldots & {w_{n} \times r_{mn} } \\ \end{array} } \right) $$

where *V* is the weighted, normalized performance matrix.

*Step 2* Calculate the values of ideal positive and negative options. These alternatives are given by^32^:

5 $$ A^{( + )} = MAX\left\{ {V_{i1} ,V_{i2} , \ldots ,V_{in} } \right\} $$

6 $$ A^{( - )} = MIN\left\{ {V_{i1} ,V_{i2} , \ldots ,V_{in} } \right\} $$

where *A*^(+)^ and *A*^(−)^ are the ideal and inferior alternatives, respectively.

*Step 3* Calculate the separation measure. Each alternative is compared to the reference points. The separation measurements can be expressed as^32^:

7 $$ S_{i}^{( + )} = \sqrt {\sum\limits_{j = 1}^{n} {\left( {V_{ij} - A^{( + )} } \right)^{2} } } $$

8 $$ S_{i}^{( - )} = \sqrt {\sum\limits_{j = 1}^{n} {\left( {V_{ij} - A^{( - )} } \right)^{2} } } $$

in which $S_{i}^{( + )}$ and $S_{i}^{( - )}$ are the separation measurements of the *i*th criterion with respect to the ideal and inferior alternatives, respectively.

*Step 4* Compute the relative closeness to the ideal solution. The relative closeness of each alternative to the ideal solution can be calculated by^32^:

9 $$ C_{i}^{(*)} = \frac{{S_{i}^{( - )} }}{{S_{i}^{( - )} + S_{i}^{( + )} }} $$

in which $C_{i}^{(*)}$ is the relative closeness of the *i*th alternative to the ideal solution ($C_{j}^{ + }$ ∈ [0, 1]). Note that a greater value of $C_{j}^{ + }$ implies the existence of more similarities between the *i*th criterion and the ideal solution.

## Results and discussions

Table 4 shows the simulated profit and employment indicators for the 13 modeling scenarios. According to the results in Table 4, the current water allocation scenario (Scenario 1) is not good in terms of profit and employment. If the goal is to increase both profit and employment in the province, Scenario 6 is the best one (i.e., 20% increase in water allocation to non-metal industry). Compared to the current water allocation scenario (Scenario 1), Scenario 6 increases the employment rate by about 14% and increases the profit by about 8.5%. That is, the best choice to increase the employment and profit is to allocate more water to the non-metal industry. In contrast, among the 13 scenarios, Scenario 7 (i.e., 20% reduction in water allocation to non-metal industry) is the worst one, in terms of these two indicators. These results demonstrate that non-metal industry is the most effective in improving the employment and profit in this province.

**Table 4:** Simulated profit index and employment index values for the 13 scenarios.

Scenario number	Employment index )person)	Profit index (billion rials)
1	64,080	1.894 × 10^7^
2	64,120	2.057 × 10^7^
3	64,020	1.730 × 10^7^
4	64,710	1.915 × 10^7^
5	63,440	1.873 × 10^7^
6	73,030	2.056 × 10^7^
7	55,120	1.732 × 10^7^
8	66,240	1.935 × 10^7^
9	61,920	1.853 × 10^7^
10	64,480	1.906 × 10^7^
11	63,670	1.882 × 10^7^
12	64,260	1.900 × 10^7^
13	63,900	1.888 × 10^7^

Table 5 shows the simulated values of three water-related indicators for the 13 scenarios, including the total water consumption of industries, the surface effluent released into the rivers, and the groundwater sustainability index (i.e., the difference between the allocated water from groundwater and the return effluent to groundwater). According to the results in Table 5, in terms of water characteristics, the basic scenario (Scenario 1) is not a suitable one, while scenario 3, which represents a 20% decrease in the first industry (chemical), has the best results for surface water and groundwater resources in dry years of water shortage. On the other hand, Scenario 10 (i.e., 20% increase in the allocated water to metal industry) is the worst scenario among the water increasing scenarios because, despite the increase in the total water allocated to industries, there are no positive changes in the values of the indicators (i.e., groundwater sustainability index and total allocated water, Table 5).

**Table 5:** Simulated water characteristics for the 13 scenarios.

Scenario number	Groundwater sustainability index (mcm)	Return of surface water (mcm)	Total allocated water (mcm)
1	0.366	0.091	5.73
2	0.418	0.101	6.05
3	0.314	0.081	5.41
4	0.367	0.092	5.85
5	0.365	0.090	5.61
6	0.371	0.093	5.92
7	0.361	0.089	5.54
8	0.375	0.094	5.95
9	0.357	0.088	5.51
10	0.370	0.094	6.01
11	0.362	0.089	5.45
12	0.367	0.091	5.75
13	0.365	0.091	5.71

In this study, the TOPSIS was used to select the best scenario based on all the defined indicators and their ranking. Table 6 shows the normalized values of the five evaluation indices for the 13 scenarios. Three of these indicators/indices (C1, C2, and C4) are positive, while the two others (C3 and C5) are negative. For the positive indicators, their increases tend to improve the region's situation, which is in line with the research goal. However, for the two negative indicators, lower values imply that they have more positive influences on the overall performance of the water allocation system.

**Table 6:** Normalized values of five indices for the 13 scenarios.

Scenario	C1	C2	C3	C4	C5
1	0.88	0.92	0.86	0.90	0.94
2	0.88	1	0.75	1	0.89
3	0.88	0.84	1	0.80	1
4	0.89	0.93	0.85	0.91	0.92
5	0.87	0.91	0.86	0.89	0.96
6	1	0.99	0.84	0.92	0.91
7	0.75	0.84	0.87	0.88	0.97
8	0.90	0.94	0.83	0.93	0.91
9	0.85	0.90	0.88	0.88	0.98
10	0.88	0.93	0.85	0.93	0.90
11	0.87	0.91	0.87	0.88	0.99
12	0.88	0.92	0.85	0.90	0.94
13	0.87	0.92	0.86	0.90	0.95

C1, C2, C3, C4, and C5 denote profit index, employment index, return of surface water, groundwater sustainability index, and total allocated water, respectively.

**Figure 6:**
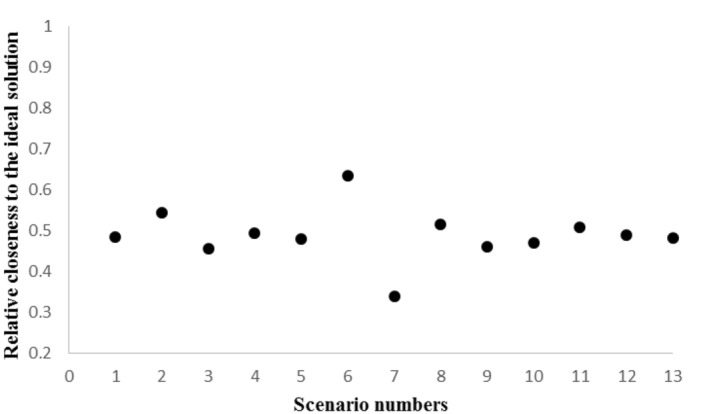
Relative closeness to the ideal solution for the 13 scenarios.

The weights of the five indices determined by using the Shannon entropy method (W1, W2, W3, W4, and W5) are 0.15, 0.23, 0.13, 0.20, and 0.27, respectively. According to these weights, it can be inferred that the fifth index (i.e., the total amount of water consumed by industries) acted as the most effective index, while the third index (i.e., return of surface water) was the least effective index. By using the TOPSIS method, the scenarios were ranked. Table 7 shows the ranks of the 13 scenarios and Table 8 displays the types of the 13 ranked scenarios (increasing or decreasing). In addition, Fig. 6 shows the relative closeness to the ideal solution for the six scenarios. According to the ranking results (Table 7), the current water allocation scenario (Scenario 1) ranked seventh among the 13 defined scenarios with a relative distance of 0.484 from the positive ideal solution. This ranking indicates that by improving the percentages of water allocated to different industries, better results can be achieved by integrating the economic, social, and environmental dimensions for the region.

**Table 7:** Ranks of the 13 scenarios based on the relative closeness to the ideal solution.

Rank	Scenario number	Relative closeness to the ideal solution
1	6	0.634
2	2	0.543
3	8	0.515
4	11	0.507
5	4	0.493
6	12	0.488
7	1	0.484
8	13	0.481
9	5	0.478
10	10	0.470
11	9	0.459
12	3	0.456
13	7	0.339

**Table 8:** Ranks and types (increasing or decreasing) of the 13 scenarios.

Rank	Scenario number	Scenario type
1	6	Increasing
2	2	Increasing
3	8	Increasing
4	11	Decreasing
5	4	Increasing
6	12	Increasing
7	1	Basic
8	13	Decreasing
9	5	Decreasing
10	10	Increasing
11	9	Decreasing
12	3	Decreasing
13	7	Decreasing

According to the analyses of the modeling results for the 13 scenarios and different industries, it was found that for chemical industry, a 20% increase in the amount of the allocated water yielded an increase of 8.5% in the profit but did not show significant changes in the employment and other indicators. For Scenario 3, it was observed that a 20% reduction in the water allocated to chemical industry led to a decrease of 8.5% in the profit and a decrease of 10% in the return of surface water. These results show the sensitivity of the profit index to the chemical industry because, with the changes in its products, there will be many changes in the economic profits of all industries. It was also found that the scenario of a 20% increase in the amount of water allocated to this group was ranked second among the scenarios. For the equipment and machinery industry, an increase of 20% in the amount of allocated water (i.e., Scenario 8) resulted in an increase of 3.3% in the employment and an increase of 2.1% in the total profit. However, it should be noted that the total amount of water consumption also increased by approximately 3.8%. Thus, if there are the necessary facilities (equipment and machinery) for the future development, Scenario 8 can be an option for water allocation. The textile industry did not show significant changes in the overall performance of the water allocation system in the region due to its small size. Similar to the textile industry, the food and beverage production group did not show significant changes in the evaluation parameters. However, the indicators of the region are very sensitive to the non-metal production and increasing or decreasing its production can cause significant changes in all five indicators. On the other hand, according to the results of the sensitivity analysis for criteria of changes of ± 10%, ± 15%, ± 20%, ± 25%, and ± 30% in the allocated water of each industry (Table 9), in special conditions when water allocation is considered as a single objective, the non-metal industry should be expanded to create more employment in the study area because this industry is the most sensitive to the employment index by changing the amount of allocated water (Table 9). The chemical industry should also be expanded to increase economic benefits because an increase of 10% in the water allocated to this industry let to an increase of 11.7% in the profit index (C2) of this industry, showing its higher sensitivity than other industries. If the object is to reduce the water allocated to the industries due to water scarcity and to ensure the sustainability of groundwater resources, reducing the water allocated to the textile industry is the best scenario (Table 9).

**Table 9:** Results of sensitivity analysis of the criteria by changing water allocations.

Industry group	Criteria	+ 10%	− 10%	+ 15%	− 15%	+ 20%	− 20%	+ 25%	− 25%	+ 30%	− 30%
Chemical	C1	0.81%	− 0.88%	1.15%	− 1.35%	1.49%	− 1.86%	1.83%	− 2.44%	2.13%	− 3.05%
	C2	11.70%	− 11.70%	17.55%	− 17.54%	23.39%	− 23.39%	29.23%	− 29.23%	35.09%	− 35.09%
	C3	5.30%	− 5.30%	7.96%	− 7.96%	10.59%	− 10.62%	13.22%	− 13.27%	15.97%	− 15.92%
	C4	− 7.16%	7.16%	− 10.74%	10.71%	− 14.32%	14.29%	− 17.87%	17.87%	− 21.45%	21.45%
Food and beverage	C1	9.78%	− 9.78%	14.67%	− 14.67%	19.56%	− 19.56%	24.45%	− 24.45%	29.34%	− 29.34%
	C2	9.76%	− 9.80%	14.64%	− 14.70%	19.61%	− 19.59%	24.49%	− 24.48%	29.37%	− 29.38%
	C3	0.54%	− 0.55%	0.81%	− 0.81%	1.08%	− 1.09%	1.35%	− 1.36%	1.63%	− 1.63%
	C4	− 0.16%	0.16%	− 0.25%	0.25%	− 0.33%	0.33%	− 0.44%	0.41%	− 0.52%	0.49%
Non-metal	C1	10.32%	− 10.32%	15.48%	− 15.48%	20.65%	− 20.65%	25.81%	− 25.81%	30.94%	− 30.97%
	C2	10.33%	− 10.33%	15.48%	− 15.49%	20.65%	− 20.65%	25.81%	− 25.82%	30.95%	− 30.98%
	C3	1.11%	− 1.12%	1.67%	− 1.68%	2.23%	− 2.23%	2.79%	− 2.79%	3.34%	− 3.35%
	C4	− 0.74%	0.74%	− 1.12%	1.09%	− 1.48%	1.48%	− 1.83%	1.83%	− 2.21%	2.21%
Equipment and machinery	C1	10.01%	− 9.96%	15.01%	− 14.97%	20.02%	− 19.96%	25.02%	− 24.97%	30.03%	− 29.97%
	C2	10%	− 10.00%	15.02%	− 14.98%	20%	− 20%	25.02%	− 24.98%	30%	− 30%
	C3	1.54%	− 1.55%	2.32%	− 2.32%	3.09%	− 3.10%	3.85%	− 3.87%	4.63%	− 4.63%
	C4	− 1.28%	1.28%	− 1.94%	1.91%	− 2.57%	2.57%	− 3.22%	3.20%	− 3.85%	3.85%
Metal	C1	7.32%	− 7.32%	10.99%	− 10.96%	14.64%	− 14.64%	18.31%	− 18.28%	21.95%	− 21.95%
	C2	8.57%	− 8.57%	12.87%	− 12.87%	17.16%	− 17.15%	21.44%	− 21.44%	25.73%	− 25.73%
	C3	− 3.27%	− 6.00%	− 2.59%	− 6.69%	− 1.91%	− 7.37%	− 1.22%	− 8.05%	− 0.54%	− 8.73%
	C4	3.28%	4.43%	2.98%	4.73%	2.68%	5.00%	2.41%	5.30%	2.10%	5.60%
Textile	C1	9.98%	− 9.98%	14.97%	− 15.08%	19.96%	− 20.07%	25.06%	− 25.06%	30.04%	− 30.04%
	C2	10%	− 10.00%	15%	− 15%	20%	− 20%	25%	− 25%	30%	− 30%
	C3	− 8.60%	− 8.86%	− 8.54%	− 8.92%	− 8.48%	− 8.98%	− 8.41%	− 9.05%	− 8.35%	− 9.11%
	C4	5.52%	5.66%	5.47%	5.71%	5.44%	5.74%	5.41%	5.79%	5.36%	5.82%

C1, C2, C3, and C4 denote profit index, employment index, return of surface water, and groundwater sustainability index, respectively.

## Concluding remarks

The current water allocation scenario (i.e., basic scenario) for the six industry groups ranked seventh out of the 13 scenarios, indicating that there were better scenarios for the region’s overall growth and development. According to the modeling and ranking of different scenarios, it was concluded that the non-metal industry had the greatest importance and desirability in improving the all-round situation of East Azarbaijan province of Iran. If the water allocated to this industry group increases by 20%, it can create more job opportunities and produce more profits than other industries in the province.

In the event of water shortage, policymakers of the water sector will have to reduce the amount of water allocated to different water consumption sectors. According to the scenarios and sensitivity analysis of different industries, a decrease in the production of the textile industry did not have significant influences on the province's employment and economic benefits. Thus, it can be selected as the best option for decision making. To reduce the amount of water consumed by the industrial sectors, the percentage of water allocated to the food and beverage industry should be reduced, considering the minimum requirements of the products of this industry for the province. Note that due to the lack of access to the data related to the equipment and technology of industries in this study, it is impossible to suggest a standard value of water consumption for any industry based on its performance. The access to such data also is crucial to performing comparison studies.

## Data Availability

The data that support the findings of this study are available from the corresponding author upon reasonable request.
